# A case report of carbamazepine‐induced oropharyngeal dysphagia in a patient with primary progressive multiple sclerosis

**DOI:** 10.1002/ccr3.7185

**Published:** 2023-05-03

**Authors:** Daniel Bishev, Mercedes Malone, Devon Best, Yvette Bazikian

**Affiliations:** ^1^ Graduate Medical Education University of Central Florida College of Medicine Orlando Florida USA; ^2^ HCA Florida North Florida Hospital Internal Medicine Residency Program Gainesville Florida USA; ^3^ HCA Florida North Florida Hospital Gainesville Florida USA

**Keywords:** carbamazepine, dysphagia, oropharyngeal, primary progressive multiple sclerosis

## Abstract

Seventy‐year‐old male with primary progressive multiple sclerosis that had a severe episode of oropharyngeal dysphagia following initiation of carbamazepine. He was being treated for trigeminal neuralgia. Four days after discontinuation of carbamazepine resulted in a complete resolution of the patient's dysphagia, and he returned to baseline.

## 1 BACKGROUND

Oropharyngeal dysphagia is a common finding in individuals with progressive neurological disease. Not only some of these individuals are more susceptible to iatrogenic causes, but they are also exposed to certain medications that may impair oropharyngeal coordination.

Certain antiepileptic drugs are documented to cause swallowing difficulties even though their effects on the nervous system do not typically cause oropharyngeal dysfunction. A study on iatrogenic causes of oropharyngeal dysphagia stated that antiseizure medications such as valproic acid, carbamazepine, and phenytoin should be used cautiously in patients at risk for dysphagia from underlying neurologic disease.^1^ Carbamazepine has been described to decrease awareness and voluntary muscle control which may affect swallowing. This is a case report about a patient who presented with oropharyngeal dysphagia that improved significantly after discontinuation of carbamazepine.

## 2 CASE REPORT

A 70‐year‐old Caucasian male with a past medical history of hypertension, hyperlipidemia, benign prostatic hyperplasia, dementia, gastroesophageal reflux disease (GERD), and primary progressive multiple sclerosis (MS) presented to the emergency department from home for evaluation of progressively worsening dysphagia of 1‐week duration. His MS was diagnosed with lumbar puncture and MRI 37 years ago and has been progressing. He was previously on dalfampridine for 3–4 years until he became bedbound 1 year prior to this admission; it was at that time that his treatment was discontinued. He has also been treated in the past with steroids for multiple MS flares. The patient's wife stated his ability to swallow food had become severely impaired over the past week. The patient frequently regurgitated and was also unable to adequately clear oral secretions on arrival.

His wife noticed an accelerated decline in his neurological status over the past year to the point that he was now bedbound, dysarthric, and intermittently cognitively impaired. Since being bed‐bound, he also developed frequent episodes of chronic urinary tract infections (UTIs). At baseline, he was conversational, able to eat soft foods, and ingest medications in pureed foods. He did not have issues with clearing secretions until about 6 days prior to admission. It was 9 days before his presentation to our facility when he was started on carbamazepine by his neurologist for the treatment of trigeminal neuralgia. The dosing was initiated at 200 mg daily for 3 days, followed by 200 mg twice daily for the 6 days leading up to the presentation.

On admission, he was noted to have altered mental status as he was only oriented to person and place but not time or situation. He was afebrile and hemodynamically stable. Physical exam was notable for crackles in the upper and lower lobes of the right lung. His laboratory testing was unremarkable except for pre‐renal acute kidney injury.

**FIGURE 1:**
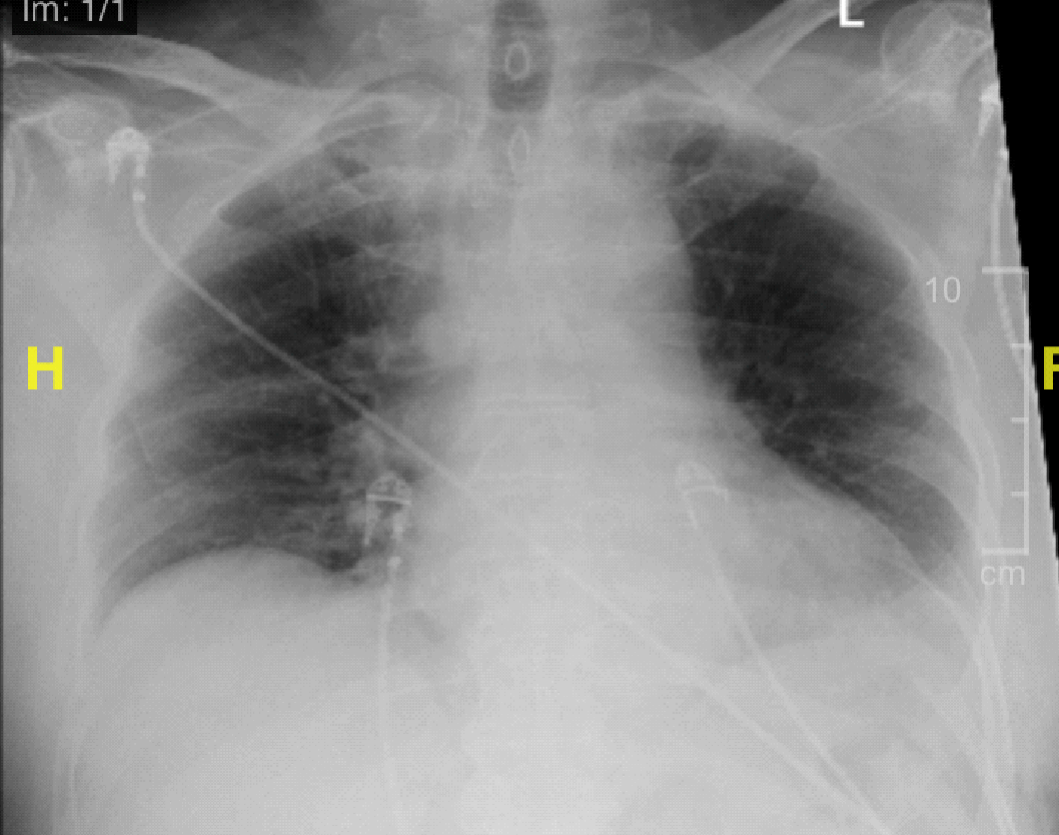
Chest X‐ray showing a right basilar opacity, possible atelectasis versus pneumonia.

**FIGURE 2:**
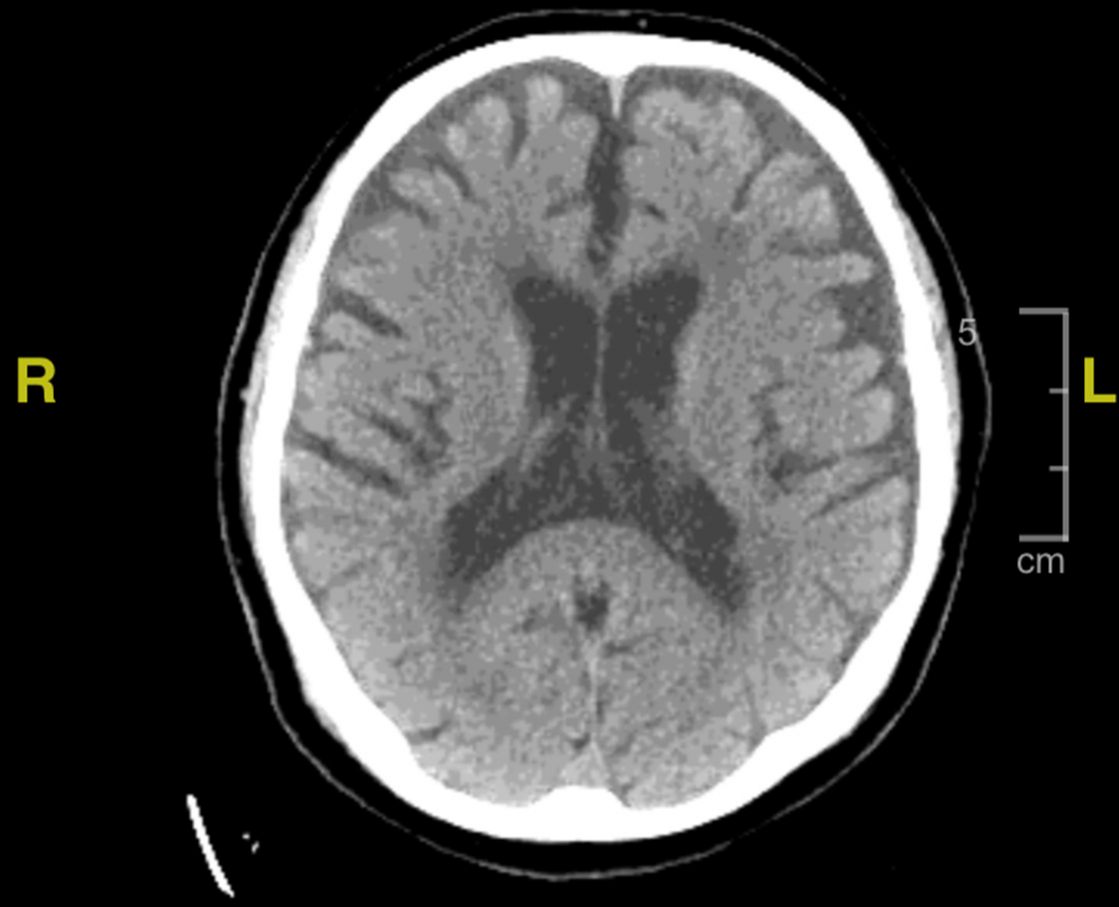
Brain CT showing no acute intracranial pathology.

**FIGURE 3:**
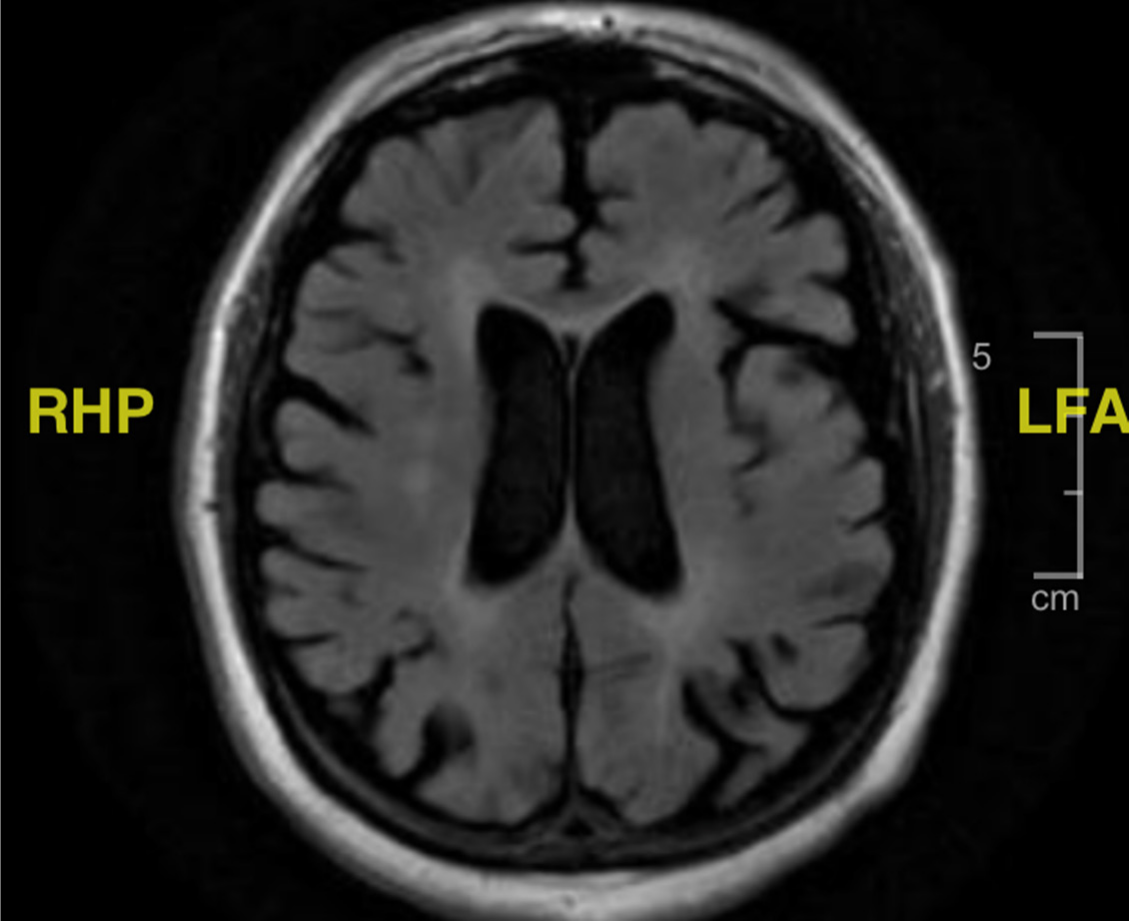
Brain MRI showing chronic periventricular demyelination and no acute intracranial pathology.

Imaging was significant for a chest x‐ray that showed a small volume of parenchymal opacity in the right lower lobe and suggested possible pneumonia (Figure 1). Brain CT did not show any new evidence for acute intracranial pathology (Figure 2). Brain MRI showed white matter disease in the periventricular areas, with no new changes in comparison to prior imaging (Figure 3). Echocardiogram showed normal systolic function, no regional wall motion abnormalities, with mild diastolic dysfunction and a moderately dilated left atrium.

During the hospitalization, he was found to have a UTI based on the results of his urinalysis. His urine culture grew extended‐spectrum beta‐lactamase *Escherichia coli*. Infectious disease was consulted, and they started the patient on intravenous piperacillin‐tazobactam 3.375 g every 8 h for 7 days followed by oral fosfomycin 3 g every 48 h for a total of 3 doses. Blood cultures collected at the time of discovering the UTI grew gram‐positive cocci in one bottle that was later determined to be a contaminant.

Due to severe dysphagia, the patient was ordered to have nothing by mouth (NPO), and a speech‐language pathologist conducted a barium‐modified swallow with fluoroscopy study. The assessment showed profound oropharyngeal dysphagia with a gross aspiration of nectar thick liquids. No cough response was appreciated during an aspiration event. The patient was unable to clear significant pharyngeal residue despite multiple swallows. He was determined to be at a severe risk for aspiration with all oral consistencies. Their recommendations were to find a permanent method of alternative feeding or to proceed with oral feeds as a means of comfort care.

Neurology was consulted to investigate reversible causes of dysphagia. Based on neurology's evaluation they did not believe that the patient's symptoms were related to an MS flare or any type of cerebrovascular event because of the gradual progression of symptoms and the remainder of the neurologic exam. They recommended discontinuing carbamazepine due to the possibility of this medication led to increased sedation with subsequent impaired neuromuscular coordination and oropharyngeal dysphagia.

Palliative care was also consulted to address the goals of care. After a discussion with the family, the decision was made to place a percutaneous endoscopic gastrostomy (PEG) tube. Thus, an interventional radiology consult was placed on day 3 of admission. Given the patient's NPO status since admission, all oral medications were held. Interventional radiology postponed placement of the PEG tube until day 4 of admission due to the active UTI and single positive blood culture, which had yet to be ruled a contaminant.

On day 4, the patient's mentation significantly improved. His secretion burden had completely resolved and his frank aspiration on oral secretions also resolved. He was able to participate in simple conversations and communicate in short sentences. Interventional radiology delayed placing the PEG tube to monitor symptom progression. On day 5, the patient's speech and enunciation continued to improve, and he began to produce a more forceful cough that allowed him to better clear secretions. Thus, a second fluoroscopic swallow study was ordered and demonstrated significant improvement in oropharyngeal dysphagia. The patient was cleared for soft pureed diet with medications administered in apple sauce. All his home medications were resumed on day 6 except carbamazepine, and he was subsequently monitored until the completion of intravenous antibiotics on day 9. The patient continued to show improvement in dysphagia, speech, and swallowing capabilities until they returned to baseline on discharge.

## 3 DISCUSSION

There are many possible precipitating factors that can lead to the development of dysphagia; some of the causes include functional or structural abnormalities of the esophagus, throat, or pharynx. The barium swallow study helped to rule out these causes. The results demonstrated impaired coordination of swallow with reduced hyolaryngeal elevation resulting in pharyngeal residue and decreased airway protection. He appears at increased risk for tracheal aspiration with thin liquids and was recommended to continue with puree texture foods. Additionally, his echocardiogram did not illustrate a severely enlarged left atrium. Cardiovascular dysphagia is another possible entity that leads to dysphagia and typically occurs when there is a mechanical obstruction by the proximity of the enlarged atrium and the esophagus.^2^

The patient had multiple overlapping conditions that may have masked the true etiology of his dysphagia. His history of GERD and MS initially obscured the diagnosis, but because of clinical suspicion and correlation of time‐course, we believe the likely trigger of his dysphagia to be carbamazepine use.

The patient's symptoms began several days after initiating carbamazepine treatment and continued to progress until its discontinuation. Stroke was ruled out, and he was only given 1 dose of 500 mg methylprednisolone in the emergency department before getting discontinued. Neurology did not consider the dysphagia to be due to MS or a cerebrovascular accident. After symptom improvement, the patient's home medications were restarted aside from carbamazepine, which did not cause a relapse in the condition. The patient had a UTI on admission, but he has been struggling with chronic UTIs for over 1 year and was asymptomatic. Dysphagia was never a presenting symptom in any of his prior UTI admissions.

Carbamazepine is known to decrease the electrochemical potential of neurons by restricting sodium influx into cells, inhibiting depolarization. It is well documented that known side effects of carbamazepine are ataxia, dizziness, or drowsiness.^3^ It can be postulated that an individual with MS is more sensitive to these types of medications due to a decrease in myelinated neurons, which can lead to motor dysfunction, particularly of the oropharynx. It is hypothesized that carbamazepine triggers impaired oropharyngeal motor coordination due to poor axonal signaling because of an underlying demyelinating condition. We also suspect that the patient may have had more profound and diffuse neuromuscular dysfunction had he not been bed‐bound and minimally mobile.

## 4 CONCLUSION

Although possibly rare, we propose that carbamazepine should be considered as a culprit for oropharyngeal dysphagia, especially in patients with underlying neurologic disease. Due to carbamazepine's ability to inhibit neuronal depolarization, it is possible that these medications can lead to increased oropharyngeal dysfunction in certain patient populations. Clinicians should be aware of the existence of carbamazepine‐induced dysphagia, especially when treating patients with progressive neurologic disease and multiple comorbidities requiring polypharmacy.

## AUTHOR CONTRIBUTIONS

**Mercedes Malone:** Conceptualization; writing – original draft; writing – review and editing. **Devon Best:** Conceptualization; writing – original draft; writing – review and editing. **Yvette Bazikian:** Writing – original draft; writing – review and editing.

## FUNDING INFORMATION

All authors have declared that they have no financial relationships at present or within the previous 3 years with any organizations that might have an interest in the submitted work.

## CONFLICT OF INTEREST STATEMENT

None.

## CONSENT

Written informed consent was obtained from the patient to publish this report in accordance with the journal's patient consent policy.

## DISCLAIMER

This work was supported by HCA Healthcare and/or an HCA Healthcare–affiliated entity. The views expressed in this publication represent those of the author(s) and do not necessarily represent the official views of HCA Healthcare or any of its affiliated entities.

## Data Availability

Data sharing not applicable to this article as no datasets were generated or analysed during the current study.
